# Global gene expression profiling in human lung cells exposed to cobalt

**DOI:** 10.1186/1471-2164-8-147

**Published:** 2007-06-06

**Authors:** Veronique Malard, Frederic Berenguer, Odette Prat, Sylvie Ruat, Gerard Steinmetz, Eric Quemeneur

**Affiliations:** 1 Service de Biochimie et Toxicologie Nucléaire, DSV/iBEB, CEA VALRHO, B.P. 17171, 30207 Bagnols-sur-Cèze, France

## Abstract

**Background:**

It has been estimated that more than 1 million workers in the United States are exposed to cobalt. Occupational exposure to ^59 ^Co occurs mainly via inhalation and leads to various lung diseases. Cobalt is classified by the IARC as a possible human carcinogen (group 2B). Although there is evidence for in vivo and in vitro toxicity, the mechanisms of cobalt-induced lung toxicity are not fully known. The purpose of this work was to identify potential signatures of acute cobalt exposure using a toxicogenomic approach. Data analysis focused on some cellular processes and protein targets that are thought to be relevant for carcinogenesis, transport and biomarker research.

**Results:**

A time course transcriptome analysis was performed on A549 human pulmonary cells, leading to the identification of 85 genes which are repressed or induced in response to soluble ^59 ^Co. A group of 29 of these genes, representing the main biological functions, was assessed by quantitative RT-PCR. The expression profiles of six of them were then tested by quantitative RT-PCR in a time-dependent manner and three modulations were confirmed by Western blotting. The 85 modulated genes include potential cobalt carriers (*FBXL2, ZNT1, SLC12A5*), tumor suppressors or transcription factors (*MAZ, DLG1, MYC, AXL*) and genes linked to the stress response (*UBC, HSPCB, BNIP3L*). We also identified nine genes coding for secreted proteins as candidates for biomarker research. Of those, *TIMP*2 was found to be down-regulated and this modulation was confirmed, in a dose-dependent manner, at protein level in the supernatant of exposed cells.

**Conclusion:**

Most of these genes have never been described as related to cobalt stress and provide original hypotheses for further study of the effects of this metal ion on human lung epithelial cells. A putative biomarker of cobalt toxicity was identified.

## Background

In the United States, more than a million workers are potentially exposed to cobalt or its compounds [1]. Cobalt is massively used in the steel industry, being a major constituent of hard metal alloys, in combination with tungsten carbides. Other industrial uses include diamond polishing with Co-containing disks and the production of drying agents, pigments, and catalysts [2]. Radioactive isotopes of cobalt are used in industry, medicine and nuclear research. In nuclear power plants, ^59^Co-containing alloys can be activated into radioactive ^60^Co oxides, dispersed in the cooling water and then contaminate workers [3,4]. A study measuring the ambient air in cobalt powder production reported concentrations of cobalt ranging from 0.675 to 10 mg/m3 [5]. Airborne concentrations measured in the working environment from a factory producing hard-metal inserts ranged from 14.6 to 37.4 mg/m3 [6]. Occupational exposure to Co occurs mainly via inhalation leading to various lung diseases, such as pneumonitis, fibrosis and asthma [7,8]. As with human exposure, animal exposure to cobalt-containing aerosols causes pronounced respiratory effects. A single 30-minute exposure of rats to relatively high levels (26–236 mg cobalt hydrocarbonyl/m3), resulted in lung congestion, oedema, and haemorrhage [9]. Necrosis and inflammation of the respiratory tract epithelium were reported in rats exposed to 19 mg cobalt/m3 and mice exposed to 1.9 mg cobalt sulfate/m3 over 16 days [10,11]. Some acute effects have been observed concerning general public exposure. Lethal cardiomyopathy was reported in people who consumed large quantities of beer containing cobalt as a foam stabilizer (0.04–0.14 mg cobalt/kg/day), and acute mortality accounted for 18% of the deaths [12]. A 19-month-old boy who swallowed an unknown amount of cobalt chloride solution died 6.5 hours after ingestion [13].

Following absorbtion by inhalation, cobalt is eliminated in the urine. Biological monitoring of accidental exposure mainly involves measuring the concentration of metal in the urine. This might be inadequate for several reasons. Firstly, the quantity of metal excreted (exposure marker) does not necessarily reflect organ damage, which varies from one person to another. Secondly, depending on the chemical form, excretion does not necessarily reflect the level of metal in the body because some forms are retained in the lungs. Thirdly, depending on its solubility, clearance can be very rapid and the cobalt may have left the body by the time samples can be taken. Therefore, a key issue in monitoring occupational exposure is the availability of adequate biomarkers.

Although the chemical toxicity of cobalt has been proven, the molecular mechanisms of its toxicity are not all known. Cobalt is genotoxic [14,15], and an oxidizing stress inducer [16]. It also induces apoptosis [17]. Cobalt is used as a hypoxia-simulating agent [18], leading to increased apoptosis, glycolysis, angiogenesis and erythropoiesis[19].

Since the lung is the main target organ of cobalt toxicity, the human A549 lung cell line was chosen as a model for this study, to evaluate cobalt toxicity. Noteworthy, this cell line has been widely documented in molecular toxicology, including hypoxia mimicked by cobalt [16,20].

Microarrays are currently used for large scale gene profiling, measuring sensitive cell changes in response to xenobiotic exposure. Such investigative studies may help identify new molecular targets for toxicants or provide new hypotheses about their mechanisms of action [21]. We used toxicogenomic tools to detect biomolecular targets of acute cobalt exposure and identify candidates as biomarkers of cobalt toxicity.

## Results and discussion

### Cobalt cytotoxicity

**Figure 1:**
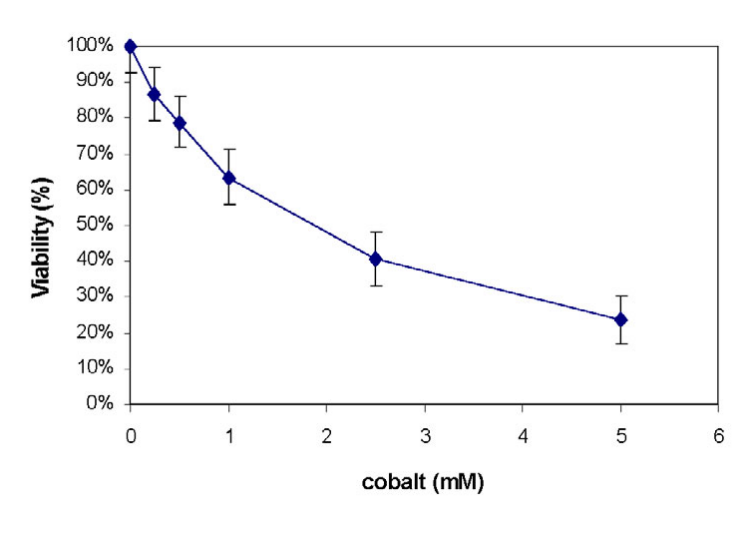
**ATP viability test on A549 cells exposed to cobalt**. The cells were cultured for 24 h with increasing concentrations of cobalt. Cell viability was determined by ATP Celltiter-Glow Luminescence Cell Viability Assay (Promega). Measurements were made on the Lumistar Galaxy (BMG) luminometer. The viability (V) was determined as the ATP ratio of treated cells versus control cells in %. The results are presented as mean ± SD (n = 4 to 8).

A549 is a stable tumor cell line, obtained from human lung carcinoma, with properties of type II alveolar epithelial cells [22]. The response of A549 cells to increasing concentrations of cobalt (CoCl2) after 24 h exposure was first analysed using the intracellular ATP measurement that reflects early metabolic modification [23]. We determined that the concentration needed to decrease ATP concentration to 50% was reached at 2 mM cobalt (figure 1). At this concentration, we measured an average load of 2.4 pg of cobalt per cell, using flame atomic absorption spectroscopy (data not shown). Some toxicologists, including one reviewer of this paper, assume that cytotoxic doses are too high to study specific cellular response to a toxicant [31]. The consequence, on gene modulation, could be the activation of large numbers of nonspecific pathways of toxicity, particularly a predominance of apoptosis and stress related genes. However, as the purpose of this study was to simulate accidental acute exposure, we nevertheless chose to expose A549 cells to an acute dose of cobalt (2 mM).

### Differential gene expression and functional classification

The global transcriptional response was monitored using CEA microarrays (GPL4263) [24]. A549 cells were treated with 2 mM cobalt in a time course experiment. For each experiment, control cells were grown in parallel and collected simultaneously. At 30 min. and 2 h, six microarrays (three dyeswaps) were hybridized with RNA from two different cell exposures. At 4 h, four microarrays (two dyeswaps) were hybridized with RNA from two exposures and at 24 h, 14 microarrays (seven dyeswaps) were hybridized with RNA from three cell exposures. Details of the experimental design and microarray data were submitted to the Gene Expression Omnibus repository at the National Center for Biotechnology Information [24] and are accessible via number GSE5892. Data analysis was performed as described in the methods section. After applying our selection criteria to the data (ratio > 1.5 and p < 0.05), we obtained a list of 173 modulated clones corresponding to 85 known genes. The modulation trend was up-regulation since 53 genes were up-regulated and 32 were down-regulated (table 1 and 2). The results showed that the main effect was obtained after 24 h of cobalt treatment (50 genes), and to a lesser extent after 30 min (16 genes). At 2 h and 4 h, 11 and 8 genes respectively were found to be modulated. Since 24 h was the main category in terms of modulated gene numbers, we reinforced the study for this time point by performing a total of 14 microarrays.

**Table 1:** Functional classification and ratios of genes up-regulated (> 1.5-fold) by exposure to cobalt across all time points

Gene ID	Gene Name	Time (h)	Microarray Ratio ^a^	qRT-PCR Ratio ^b^
*Development, differentiation and proliferation*				
***DNAJA2***	**DnaJ (Hsp40) homolog, subfamily A, member 2**	**24**	**1.94**	2.54 *
***OAZ1***	**ornithine decarboxylase antizyme 1**	**24**	**1.68**	2.26 *
***PGF***	**placental growth factor, vascular endothelial growth factor-related protein**	**0.5**	**1.50**	1.37*
***ST13***	**suppression of tumorigenicity 13 (colon carcinoma) (Hsp70 interacting protein)**	**24**	**1.52**	1.59 *
***TNFRSF9***	**tumor necrosis factor receptor superfamily, member 9**	**0.5**	**1.55**	0.54 *
*Signal transduction and trafficking*				
*CTNNB1*	catenin (cadherin-associated protein), beta 1, 88 kDa	24	1.73	
***DLG1***	**discs, large homolog 1 (Drosophila)**	**24**	**3.42**	0.38 ***
***GNB2L1***	**guanine nucleotide binding protein (G protein), beta polypeptide 2-like 1**	**24**	**1.74**	2.64 *
*PLA2R1*	phospholipase A2 receptor 1, 180 kDa	0.5	1.52	
*SCAMP1*	secretory carrier membrane protein 1	24	4.00	
***SLC12A5***	**solute carrier family 12, (potassium-chloride transporter) member 5**	**0.5**	**1.58**	1.69 ***
***SLC25A6***	**solute carrier family 25 (mitochondrial carrier; adenine nucleotide translocator), member 6**	**24**	**1.89**	3.12 ***
***SLC2A1***	**solute carrier family 2 (facilitated glucose transporter), member 1**	**24**	**1.85**	3.41 *
*SLC6A11*	solute carrier family 6 (neurotransmitter transporter, GABA), member 11	0.5	1.54	
*STXBP1*	syntaxin binding protein 1	0.5	1.53	
*Cell defense*				
***BNIP3L***	**BCL2/adenovirus E1B 19 kDa interacting protein 3-like**	**24**	**1.89**	3.30 *
*CNIH*	cornichon homolog (Drosophila)	24	1.62	
***IFNA4***	**interferon, alpha 4**	**0.5**	**1.60**	0.32 *
*MGST1*	microsomal glutathione S-transferase 1	24	3.80	
***SERPINC1***	**serine (or cysteine) proteinase inhibitor, clade C (antithrombin), member 1**	**24**	**1.59**	5.02 *
*Protein expression and turn-over*				
*CANX*	calnexin	24	1.52	
*EEF1A1*	eukaryotic translation elongation factor 1 alpha 1	24	1.63	
*EEF1G*	eukaryotic translation elongation factor 1 gamma	24	1.76	
*EIF4A1*	eukaryotic translation initiation factor 4A, isoform 1	24	1.64	
*HSPA1A*	heat shock 70 kDa protein 1A	24	3.22	
***HSPCB***	**heat shock 90 kDa protein 1, beta**	**24**	**2.21**	2.51 *
*KIAA1940*	KIAA1940 protein	2	2.09	
*PPIA*	peptidylprolyl isomerase A (cyclophilin A)	24	1.59	
*RPL15*	ribosomal protein L15	24	1.73	
*RPL35A*	ribosomal protein L35a	24	1.68	
*RPL36AL*	ribosomal protein L36a-like	24	1.55	
*SERPINH1*	serine (or cysteine) proteinase inhibitor, clade H (heat shock protein 47), member 1,	24	1.61	
*SUI1*	putative translation initiation factor	24	2.56	
*UBA52*	ubiquitin A-52 residue ribosomal protein fusion product 1	4	2.22	
*UBB*	ubiquitin B	24	1.94	
***UBC***	**ubiquitin C**	**24**	**5.58**	8.17 *
*WFDC2*	WAP four-disulfide core domain 2	24	1.56	
*Gene transcription and modification*				
***BHC80***	**BRAF35/HDAC2 complex (80 kDa)**	**24**	**1.76**	1.85 *
***HOXA9***	**homeo box A9**	**0.5**	**1.50**	2.02 *
*SDCCAG33*	biglycan; serologically defined colon cancer antigen 33	0.5	1.51	
*Cell structure and mobility*				
*SDC3*	syndecan 3 (N-syndecan)	24	1.62	
*Metabolism and energy*				
*AGPAT3*	1-acylglycerol-3-phosphate O-acyltransferase 3	24	1.64	
*ALDOA*	aldolase A, fructose-bisphosphate	24	2.75	
*GAPD*	glyceraldehyde-3-phosphate dehydrogenase	24	3.01	
*LDHA*	lactate dehydrogenase A	24	1.66	
*OAT*	ornithine aminotransferase (gyrate atrophy)	24	3.15	
*PGK1*	phosphoglycerate kinase 1	24	1.77	
*SMAP1*	stromal membrane-associated protein 1	24	1.65	
*TKT*	transketolase (Wernicke-Korsakoff syndrome)	24	1.86	
*Unknown function*				
*GPM6A*	glycoprotein M6A	2	1.62	
*LOC115704*		24	1.63	
***LTBP3***	**latent transforming growth factor beta binding protein 3**	**2**	**1.71**	2.37 *

a: p value < 0.05 with 1.5 fold change.

b: validation with quantitative qRT-PCR (lines in bold) :

*: p value < 0.005, ** p value < 0.01, *** p value < 0.05.

Microarray p-values were calculated using a t-test statistical analysis (Benjamini-Hochberg FDR) on Genespring software. Pair Wise Fixed Reallocation Randomized Test was used to calculate qRT-PCR p values with REST software.

**Table 2:** Functional classification and ratios of genes down-regulated (< 1.5 fold) by exposure to cobalt across all time points

Gene ID	Gene Name	Time (h)	Microarray Ratio ^a^	qRT-PCR Ratio ^b^
*Development, differentiation and proliferation*				
***NDRG4***	**NDRG family member 4**	**24**	**0.58**	0.29 ***
*SEZ6L*	seizure related 6 homolog (mouse)-like	4	0.62	
*Signal transduction and trafficking*				
*ATP6V1A*	ATPase, H+ transporting, lysosomal 70 kDa, V1 subunit A	2	0.66	
***AXL***	**AXL receptor tyrosine kinase**	**4**	**0.60**	1.36 ***
***GFRA2***	**GDNF family receptor alpha 2**	**0.5**	**0.63**	0.09 *
*ICAM1*	intercellular adhesion molecule 1 (CD54), human rhinovirus receptor	2	0.60	
***IFNAR2***	**interferon (alpha, beta and omega) receptor 2**	**24**	**0.61**	0.55 ***
***MAPK10***	**mitogen-activated protein kinase 10**	**24**	**0.47**	0.31 ***
*S*100*A*4	S100 calcium binding protein A4	2	0.64	
***TFRC***	**transferrin receptor (p90, CD71)**	**0.5**	**0.50**	0.65 *
*Cell defense*				
***BAG1***	**BCL2-associated athanogene**	**2**	**0.65**	0.55 **
*TNFSF6*	tumor necrosis factor (ligand) superfamily, member 6	4	0.54	
*Protein expression and turn-over*				
*CNDP2*	CNDP dipeptidase 2 (metallopeptidase M20 family)	4	0.57	
***FBXL2***	**F-box and leucine-rich repeat protein 2**	**0.5**	**0.55**	0.75 ****
***TIMP2***	**tissue inhibitor of metalloproteinase 2**	**24**	**0.63**	0.54 ***
*Gene transcription and modification*				
***MAZ***	**MYC-associated zinc finger protein (purine-binding transcription factor)**	**2**	**0.28**	0.59 **
*NT5E*	5'-nucleotidase, ecto (CD73)	24	0.50	
*PAI-RBP1*	PAI-1 mRNA-binding protein	2	0.62	
*SFRS1*	splicing factor, arginine/serine-rich 1 (splicing factor 2, alternate splicing factor)	0.5	0.52	
***SIN3A***	**SIN3 homolog A, transcriptional regulator (yeast)**	**24**	**0.63**	2.21 *
*Cell structure and mobility*				
*FAT2*	FAT tumor suppressor homolog 2 (Drosophila)	4	0.56	
*TUBB2*	tubulin, beta, 2	4	0.62	
*VIL2*	villin 2 (ezrin)	0.5	0.64	
*Metabolism and energy*				
*AKR1C3*	aldo-keto reductase family 1, member C3	24	0.53	
*ALDH1A1*	aldehyde dehydrogenase 1 family, member A1	24	0.60	
*GLCE*	glucuronyl C5-epimerase	0.5	0.46	
*GPD2*	glycerol-3-phosphate dehydrogenase 2 (mitochondrial)	4	0.55	
*MTHFS*	5,10-methenyltetrahydrofolate synthetase	2	0.52	
*Unknown function*				
*C20orf30*	chromosome 20 open reading frame 30	2	0.48	
*C20orf30*	chromosome 20 open reading frame 30	24	0.60	
*FLJ12806*	hypothetical protein FLJ12806	0.5	0.64	
*KIAA1582*	KIAA1582 protein	24	0.58	

a: p value < 0.05 with 1.5 fold change.

b: validation with quantitative qRT-PCR (lines in bold) :

*: p value < 0.005, ** p value < 0.01, *** p value < 0.05, **** p value < 0.1.

Microarray p-values were calculated using a t-test statistical analysis (Benjamini-Hochberg FDR) on Genespring software. Pair Wise Fixed Reallocation Randomized Test was used to calculate qRT-PCR p values with REST software.

We used DAVID [25] web-accessible software, to obtain information from the Gene Ontology Database. We then sorted the modulated genes manually into 8 major functional classes (table 1 and 2), combining information from DAVID, SWISS-PROT and additional elements taken from the literature. The classifications of genes modulated after 30 min and 24 h of cobalt treatment were compared. At 30 min, the main category regulated was signal transduction and trafficking (38%) and half of the modulated genes coded for membrane proteins. This suggests strong involvement of membrane transport in the early response to cobalt exposure. The later response (24 h) mainly concerned protein expression and turnover (34%), metabolism and energy (20%), and finally signal transduction and trafficking (16%). This different pattern indicates that after 24 h, the cell response is established, with metabolic adaptation and strong involvement of protein translation. It can be noticed that in spite of the cytotoxic dose we used, the genes modulation trend was rather increase than decrease, indicating an active metabolic response of the cell, and also that apoptotic and stress related genes are not over represented.

### Quantitative RT-PCR

**Figure 2:**
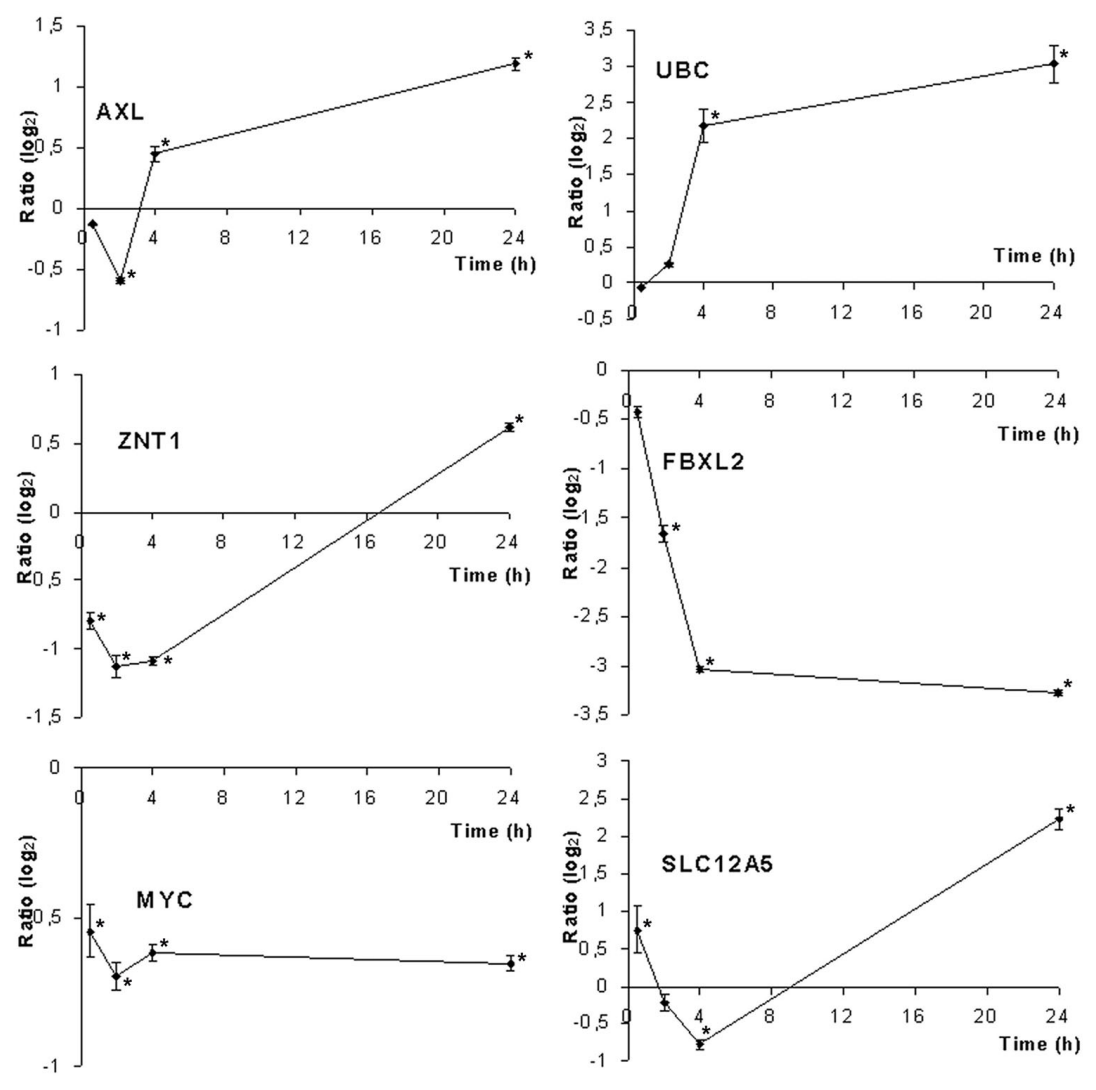
**Time-course qRT-PCR analysis**. The temporal expression pattern of genes regulated by cobalt was analysed by qRT-PCR in sextuplets for each time point. Mean results are given in Log2 ratios ± SD. *: significant modulation calculated by pairwise testing with p < 0.05

We selected genes to be validated by quantitative RT-PCR (qRT-PCR) in some specific functional classes: development, differentiation and proliferation, signal transduction and trafficking, cell defense and finally gene transcription and modification. We also selected genes coding for secreted proteins as potential hits for biomarker research. Tables 1 and 2 show the results of quantitative RT-PCR validation (lines in bold) and the primers used are described in table 3. Of the 29 genes tested, most confirmed the variation observed in the microarrays (83%). Five genes appeared to be modulated in the opposite direction by qRT-PCR (*AXL, IFNA4, DLG1, TNFRSF9 *and *SIN3A*). Some discrepancies between the two techniques may be due to technical artefacts even if the primers were designed within the microarray cDNA sequence. For further analysis of gene modulation, we performed time-course quantitative RT-PCR on several genes: *AXL, UBC, FBXL2 *and *SLC12A5 *(figure 2). The results show that modulation varies over time following various profiles: either diminishing at first, then increasing up to 24 h (*AXL, SLC12A5*), or being induced from 2 h (*UBC*), or finally, early and continuous diminishing (*FBXL2*). For genes displaying temporal modulation, down-fluctuation quickly followed by up-regulation, as with *AXL *(figure 2), a slight difference in the kinetics of different exposures could explain the opposing results we found between microarray and qRT-PCR ratios, because RNAs from different exposures were used to perform the microarray experiments and qRT-PCR. These results, revealing an oscillatory gene expression profile, suggest complex regulation pathways in response to cobalt.

**Table 3:** List of primers used in qRT- PCR

Gene ID	Gene Name	GenBank ID	Forward primer	Reverse primer	Amplicon (bp)
*AXL*	AXL receptor tyrosine kinase	NM_001699	GACCGGCCAAGTTTTACAGA	ATAACCTCCACCCTCATCCA	117
*BAG1*	BCL2-associated athanogene	NM_004323	GCAGCAGTGAACCAGTTGTC	CGGTGTTTCCATTTCCTTCA	119
*BHC80*	BRAF35/HDAC2 complex (80 kDa)	NM_016621	CCGAGCCGTTTGTTTAGGTA	CACTGGGGTTGGTGAAATCT	117
*BNIP3L*	BCL2/adenovirus E1B 19 kDa interacting protein 3-like	NM_020221	ATGTTTGGCTTTGGGGCTA	CTTCACAGGTCACACGCATT	109
*DLG1*	discs, large homolog 1 (Drosophila)	NM_004087	CAGCCAGATACTCCCCAGTT	TGAGCCACGATGAAGAACAA	89
*DNAJA2*	DnaJ (Hsp40) homolog, subfamily A, member 2	NM_005880	CCAGGGTGTGTTCGTGTAGTT	TGGGTTGATCCAGTTGTTTTC	120
*FBXL2*	F-box and leucine-rich repeat protein 2	NM_012157	CAGAACTGCCGAAACATTGA	CACACAGGAGGTCAGATCCA	123
*FIGF*	c-fos induced growth factor (vascular endothelial growth factor D)	NM_004469	GAACACCAGCACCTCGTACA	TGGCAAGCACTTACAACCTG	118
*GFRA2*	GDNF family receptor alpha 2	NM_001495	GAGACACACGGTCACTGGAA	TCGAGGACGAGAGACTGGAG	126
*GNB2L1*	guanine nucleotide binding protein (G protein), beta polypeptide 2-like 1	NM_006098	GGTGTCTTGTGTCCGCTTCT	CAATGTGGTTGGTCTCAGC	119
*HOXA9*	homeo box A9	NM_152739	CACCAGACGAACAGTGAGGA	ACTCCGTTACAATCAGCATTCA	111
*HSPCB*	Heat shock protein HSP 90-beta	NM_007355	GGAGAGGAGGAGGTGGAGAC	GAGGGTTGGGGATGATGTC	217
*IFNA4*	interferon, alpha 4	NM_021268	GAAGAAATACAGCCCTTGTGC	TGAACCAGTTTTCAATCCTTCC	114
*IFNAR2*	interferon (alpha, beta and omega) receptor 2	NM_000874	GTCTCGCTAAGGGCTGGAAT	AGGCAGGACGACTGTTTGAG	94
*LTBP3*	latent transforming growth factor beta binding protein 3	NM_021070	CCAGGGCTACAAGAGGCTTA	GGCAGACACAGCGATAGGAG	119
*MAPK10*	mitogen-activated protein kinase 10	NM_002753	CTTCCCAGATTCCCTCTTCC	GTAAGGCGTCGTCCACTGAT	128
*MAZ*	MYC-associated zinc finger protein (purine-binding transcription factor)	NM_002383	CGGATCACCTCAACAGTCAC	ATGGCACTTTCTCCTCGTGT	135
*MYC*	v-myc myelocytomatosis viral oncogene homolog (avian)(MYC)	NM_002467	AAAGGCCCCCAAGGTAGTTA	TTTCCGCAACAAGTCCTCTT	103
*NDRG4*	NDRG family member 4	NM_020465	ATGCTTTCCATCCACTCACC	TTCACTGCTCTCTCCCGTTT	115
*OAZ1*	ornithine decarboxylase antizyme 1	NM_004152	GAGCCGACCATGTCTTCATT	CCCGGTCTCACAATCTCAAA	100
*PGF*	placental growth factor, vascular endothelial growth factor-related protein	NM_002632	ACCCCTTGGAGGAGAGAGAC	GCATTCAGCAGGGAAACAGT	119
*SERPINC1*	serine (or cysteine) proteinase inhibitor, clade C (antithrombin), member 1	NM_000488	CAATCGCCTTTTTGGAGACA	TGGACACCCATTTGTTGATG	146
*SIN3A*	SIN3 homolog A, transcriptional regulator (yeast)	NM_015477	CTCCCAACTGCAAGCACATA	TCCCAACGAGATTGTCACTG	115
*SLC12A5*	solute carrier family 12, (potassium-chloride transporter) member 5	NM_020708	CAAGGGTCCAACTTTTCCTG	GCCTCTCGGTTTCTTCCTCT	152
*SLC25A6*	solute carrier family 25 (mitochondrial carrier; adenine nucleotide translocator), member 6	NM_001636	CTGTTTTGCACAGCCGAGTA	TTTTGACCTCTGCGTCCTCT	87
*SLC2A1*	solute carrier family 2 (facilitated glucose transporter), member 1	NM_006516	GTGGAGACTAAGCCCTGTCG	CATAGCCACCTCCTGGGATA	128
*ST13*	suppression of tumorigenicity 13 (colon carcinoma) (Hsp70 interacting protein)	NM_003932	AGGCAGACGAACCATCAAGT	TCCGTTATCTCCGCATTTTC	115
*TFRC*	transferrin receptor (p90, CD71)	NM_003234	CGCTGGTCAGTTCGTGATTA	TCAGGCCCATTTCCTTTATG	134
*TIMP2*	tissue inhibitor of metalloproteinase 2	NM_003255	TTCATTCGTCTCCCGTCTTT	ACCAACGTGTGTGGATCAAA	113
*TNFRSF9*	tumor necrosis factor receptor superfamily, member 9	NM_001561	AGGGCTGTTGGGACTTTCTT	GGATGGTGTTCTTGCTTTTGA	83
*TUBA3*	tubulin, alpha 3	NM_006009	CCTACAACTCCATCCTCACCA	GTCAACATTTCAGGGCTCCA	203
*UBC*	ubiquitin C	NM_021009	GGAACAGGCGAGGAAAAGTA	AACAAGAACTGCGACCCAAA	146
*ZNT1*	solute carrier family 30 (zinc transporter), member 1	NM_021194	ACCCAGAAAACCCCAGAAGT	CACTGAACCCAAGGCATCTC	158

### Hypoxia

Cobalt is known as a "hypoxia-simulating" agent[26] and most of the literature therefore relates to this property. Hypoxia-inducible factor (HIF-1) is a transcription factor which acts as the main regulator of oxygen homeostasis and the genes it induces have mostly been described [27]. We compared this list with our results to identify modulations due to hypoxia. Strikingly, very poor overlap was observed, as only 7 of the 85 modulated genes (8 %) in our study matched HIF1 target genes. These 7 genes are: aldolase A (*ALDOA*), glucose transporter 1 (*SLC2A1*), glyceraldehyde 3-phosphate dehydrogenase (*GAPD*), lactate dehydrogenase A (*LDHA*), proapoptotic factor (*BNIP3L*), phosphoglycerate kinase 1 (*PGK1*) and transferrin receptor (*TFRC*), (table 1 and 2). Like HIF1 regulated genes, they were all induced at 24 h by cobalt (except *TFRC *which was repressed at 30 min), therefore our results agree with those already published and validate our system relative to previous reports, since they represent an appropriate internal positive control. Recently, two transcriptomic studies of hypoxia compared the action of low oxygen with that of metal ions such as cobalt or nickel on embryonic mouse fibrobasts [28] and human liver carcinoma [29]. 21% and 28% of the genes respectively were found to be modulated by both hypoxia and cobalt. This shows that the toxic effect of cobalt is not limited to hypoxia. It should also be noted that nickel had an action closer to that of low oxygen than cobalt, with 65% common modulated genes [29]. Comparing these results with ours showed that, apart from the hypoxia genes, we had no overlap. This discrepancy might be due to differences between cell models, species and conditions of exposure (one non-lethal dose of 0.1 mM cobalt for 24 h).

### Stress response – apoptosis

**Figure 3:**
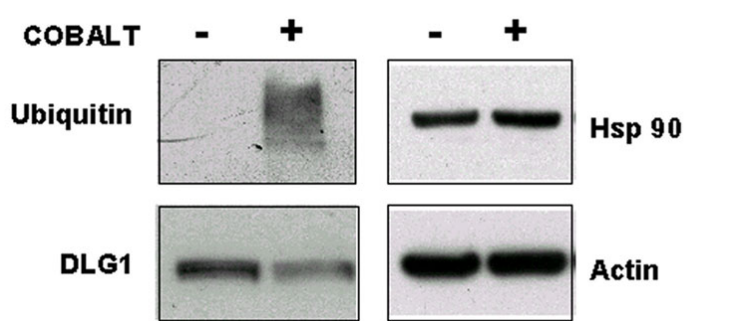
**Western blot confirmation**. Western blot analyses were performed on A549 extracts following exposure to 2 mM cobalt for 24 h. Treated (Co+) and control (Co-) extracts were probed with anti-ubiquitin, anti-DLG1, or anti-HSP90 antibodies. An anti-actin antibody was used as a control.

The *HSPCB *gene coding for heat shock protein 90, was induced by a factor of 2.2 at 24 h (table 1). HSP90 protein plays a major role in the stress response by preventing irreversible protein aggregation. In our previous study of the toxicogenomics of uranium [30], we noted that both *HSPCB *gene and HSP90 protein were strongly repressed. This protein can even be thought of as a metal "sensor", since the *HSPCB *gene was also reported to be modulated by other metals (Ni, As, Cr, Cd) [31]. However, we did not see any change in the quantity of HSP90 in Western blotting experiments (figure 3). This proteome/transcriptome discordance can be explained by an increase in protein turnover associated with gene induction, resulting in a constant amount of protein being maintained in the cell. Another explanation may be that, as Gerner observed for apoptosis, HSP90 is aggregated and accumulates in the insoluble fraction of the cytosol and is not collected during protein extraction [32]. Two other genes described as HSP mediators are also induced by cobalt: *ST13 *and *DNAJA2*.

Ubiquitin is a key element in eliminating proteins via proteasome because polyubiquitinylation is the recognition signal for protein elimination [33,34]. The results show that ubiquitin genes were up-regulated after 24 h of toxic exposure (see table 1, *UBB*, ratio 1.94, *UBC*, ratio 5.6) while *UBC *gene induction was observed in the kinetics from 4 h with qRT-PCR (figure 2), showing that it is not an early event in cell response. The Western blot revealed protein ubiquitinylation, with the anti-ubiquitin antibody, shown by a smear at high molecular weight in the cobalt extract, indicating an accumulation of ubiquitinylated protein (figure 3). This demonstrates increased turnover of proteins involved in defense against stress, or the elimination of proteins partly damaged by the metal, or the start of apoptosis.

Cobalt has been described as an apoptosis inducer [17] and this is confirmed here by *BAG1 *repression and *BNIP3L *induction (table 1 and 2).

### Tumor suppressors – transcription factors

The protein encoded by *MAZ *(Myc-associated zinc finger protein), is a transcription factor involved in both the initiation and termination of target gene transcription. It binds zinc and can act as a tumor suppressor [35]. This gene is repressed early at 2 h. Other cobalt-modulated genes code for zinc-binding proteins involved in transcription or DNA metabolism: *BHC80 *(BHC80 protein), *NT5E *(CD73) and *SDCCAG33 *(Teashirt homolog 1). Cobalt ions have been shown to substitute for zinc in zinc finger protein domains which control the transcription of several genes and also in zinc-DNA repair proteins, inhibiting DNA repair [14,36,37].

*DLG1 *(disk large homolog 1) has been identified as a tumor suppressor gene in drosophila, with its mutation inducing the loss of cell polarity and neoplastic tissue growth. This gene is highly conserved. The molecular mechanism which regulates cell proliferation by DLG1 is not well known but there are arguments for implicating epithelial cell polarization in this regulation [38]. The *DLG1 *(disk large homolog 1) gene was strongly repressed in qRT-PCR. The anti-DLG1 antibody revealed clear depletion of DLG1 protein in Co+ samples (figure 3). This confirmed the results of the qRT-PCR.

Two tumor suppressors (*MAZ *and *DLG1*) were repressed by cobalt, and in one case (*DLG1*), this repression was also observed at protein level.

We noted the modulation of three genes related to *MYC*, a key element in oncogenic response (table 1 and 2). The first, *SIN3A*, known as an *MYC *suppressor, is induced by cobalt. The two others: *NDRG4*, a gene negatively regulated by *MYC *and *MAZ*, a *MYC *transcription inhibitor, are down-regulated by cobalt. These results are not clear regarding *MYC *up- or down-regulation, but *MYC *regulation pathways are complex. These results led us to hypothesize that *MYC *might be modulated by cobalt, so we tested *MYC *temporal modulation with qRT-PCR. Interestingly, we noted significant *MYC *inhibition from 30 min to 24 h (figure 2) indicating that cobalt modulates regulation pathways controlling or controlled by *MYC*. Liu has recently shown that *MYC *is involved in the carcinogenic response of mouse liver to arsenic, another toxic metal [39].

The *AXL *gene is positively modulated. Members of the AXL/UFO family of tyrosine kinases are prone to transcriptional regulation and perform various functions including regulation of cell adhesion, migration, phagocytosis, and survival. The biological consequences of *AXL *activation are complex. *AXL *was initially identified as a transforming gene product, and AXL expression is indeed up-regulated in human tumors [40,41].

Concerning the potential effects of cobalt on cancer development, it will be interesting to carry out further studies of the mechanisms by which cobalt induces these responses. These genes have never been linked to cobalt effects before.

### Transporters

Two cobalt transporters are described in yeast: *COT1 *and *COT2 *[42]. Eleven cobalt-tolerant mutants were obtained with a common *COT2 *mutation, suggesting that it is involved in cobalt uptake [43]. The mutants were resistant to cobalt toxicity and cobalt was not incorporated in yeast cells. The human homolog of *COT2, FBXL2*, was strongly repressed in our study as early as 2 h and with a ratio of 0.1 at 24 h; this is the strongest inhibition observed in time-course qRT-PCR analysis (figure 2). FBXL2 function is not very well described, but it is potentially involved in ubiquitinylation. Conklin's observations on the yeast homolog of *FBXL2 *[43] and our results, lead us to suggest that *FBXL2 *gene repression could be a cellular defense mechanism and this protein might be associated with cobalt uptake in humans.

The product of the *COT1 *gene is involved in the uptake of cobalt ions [44]. *COT1*, has a human homolog in *ZNT1*, coding for Zinc transporter 1 protein, probably involved in zinc transport out of the cell. *ZNT1*, not spotted on the microarray, was also tested in kinetics with qRT-PCR and was found to be slightly modulated with a biphasic response; first down- then up-regulated (figure 2).

The *SLC12A5 *gene, coding for an integral membrane K-Cl cotransporter, was repressed at 4 h then strongly increased at 24 h (qRT-PCR ratio : 4.3). The three genes, *FBXL2, ZNT1 *and *SLC12A5*, are therefore appropriate candidates for research into cobalt transport proteins in human cells and will be studied further using targeted biological approaches.

### Potential biomarkers and secreted proteins

**Figure 4:**
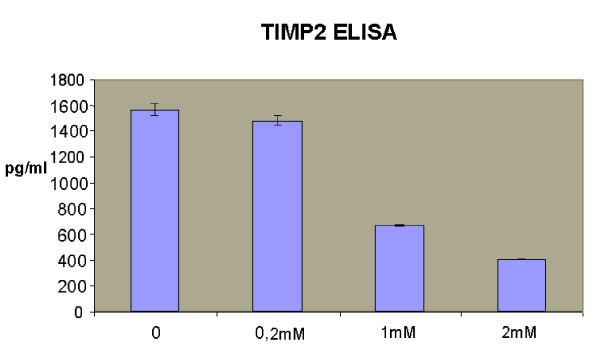
**TIMP2 ELISA**. Concentrations of TIMP2 in supernatants were determined by ELISA following treatment of A549 cells with cobalt over 24 h. The changes in level of TIMP2 in cell supernatants between cobalt and the control were -5% for 0.2 mM, -57% for 1 mM and -72% for 2 mM (n = 2).

Biological monitoring of exposure to cobalt is mainly based on its concentration in urine. This method may be inadequate for two reasons. Firstly, measuring the quantity of excreted metal (exposure marker) does not necessarily reflect organ damage, which varies from one person to another. Furthermore, depending on the chemical form, excretion does not necessarily reflect the concentration of metal in the body; indeed, certain forms are eliminated in several phases; only 40% of cobalt oxides inhaled are excreted within 72 h [45]. In this context, only an effective biomarker could evaluate the damage and provide a valuable tool for monitoring occupational contamination in the event of accidental acute exposure or chronic exposure. We selected nine modulated genes coding for secreted proteins: *ICAM1 *(CD54), *SERPIN C1 *(antithrombin-III), *IFNA4 *(alpha-4 interferon), *TFRC *(transferrin receptor), *PLA2R1 *(phospholipase receptor A2), *TNFSF6 *(FAS ligand), *WFDC2 *(WAP four-disulfide core domain protein 2), *LTBP3 *(latent transforming growth factor beta binding protein 3) and *TIMP2 *(tissue inhibitor of metalloproteinase 2). To further evaluate the interest of these genes, we checked the secretion modulation of the corresponding proteins. A549 cells were exposed to cobalt in a dose-response manner, to 0.2, 1 and 2 mM, and the supernatants analysed using available immunoassays. The levels of FAS ligand, WFDC2 [46], ICAM1, antithrombin-III, alpha-4 interferon, and soluble transferrin receptor were found to be invariant or below the kit detection limit. These results indicate that most of these immunoassays were not sensitive enough to detect faint modulations. TIMP2 protein (tissue inhibitor of metalloproteinase 2) was measured using ELISA and we confirmed the modulation recorded at gene level (microarray ratio: 0.63, qRT-PCR ratio: 0.69). A strong decrease in TIMP2 supernatant concentration was noted at 1 or 2 mM of cobalt (figure 4). The changes in TIMP2 levels in cell supernatants between cobalt and the control were -5% for 0.2 mM, -57% for 1 mM and -72% for 2 mM. The reason for this reduction in TIMP2 can be explained as a consequence of cobalt exposure. Matrix metalloproteinases (MMPs) are a family of endopeptidases that can degrade all the components of the extracellular matrix [47]. Endogenous protease inhibitors, known as tissue inhibitors of metalloproteinase (TIMP), provide critical extracellular regulation of MMP proteolytic activities [48,49], regulating tumor growth, progression, and angiogenesis in a variety of experimental cancer models and human malignancies. *TIMP2 *mRNA is described as being suppressed by hypoxia in endothelial cells [50]. Exposure of cells to cobalt up-regulates indirectly hypoxia-inducible genes by a mechanism which has recently been explained [51]. Therefore, TIMP-2 down-regulation might be a consequence of cobalt-simulated hypoxia. Down-regulation of TIMP2 mediated by ROS production has been observed by Rezzani in rat cardiac tissue exposed to cyclosporin [52]. Since it is known that cobalt triggers ROS production [16], TIMP2 modulation by cobalt could also be explained by this pathway.

TIMP2 protein modulation in cobalt-exposed cell supernatant corroborated the gene down-regulation observed on the microarrays and with qRT-PCR. TIMP2 modulation has never been associated with metal stress. This result is innovative and will be studied further in the biological fluids of rats exposed to different chemical forms of cobalt by inhalation.

## Conclusion

This study provides the first toxicogenomic analysis of human lung cell response to acute cobalt exposure. We have confirmed that genes involved in the cobalt hypoxia response and apoptosis are modulated. We have also revealed genes linked to heat shock response and proteasome function that have already been described in other metal stress responses. Newly identified genes linked to cobalt acute toxicity include potential cobalt carriers (*FBXL2, ZNT1, SLC12A5*) and tumor suppressors or transcription factors (*MAZ, DLG1, MYC, AXL*). Some of these genes provide new hypotheses for elucidating the mechanisms of cobalt intracellular chemical toxicity. Targeted biological approaches might confirm their biochemical role in the cobalt response.

Regarding biomarkers, we have highlighted the down-regulation of *TIMP2*, a gene coding for a secreted protein. TIMP2 modulation was confirmed at protein level, in a dose-dependent manner, in the supernatant of exposed A549 cells. TIMP2 provides a putative biomarker of cobalt toxicity that will be further studied on animal models.

## Methods

### Cell culture

The human type II epithelial cell line A549 from ATCC, was cultured as described previously [53]. At midlog phase, a medium without FCS, containing cobalt (CoCl2, Sigma) or not, was added for 24 hours. A CoCl2 stock solution (1 M in MilliQ water) was prepared extemporaneously, filtered through a sterile 0.22 μm membrane and then diluted in the cell culture medium (final concentration 2 mM). After 30 mn, 2 h, 4 h or 24 h, the cells were harvested with trypsin and washed in PBS containing 1 mM EDTA. Culture supernatants for ELISA and cells for Western blot confirmations were collected after 24 h of exposure.

### Cytotoxicity studies

Cobalt cytotoxicity was determined by measuring the intracellular ATP after 24 h using the Promega Celltiter-GlowTM Luminescence Cell Viability Assay (four to eight replicates per concentration). The viability rate was determined as the ratio between the ATP in treated cells and control cells. Cobalt concentration in cell pellets was measured using flame atomic absorption spectroscopy (FAAS, CERECO laboratory, Nimes, France).

### RNA extraction

Total RNA was isolated using the Quiagen RNeasy miniprep kit according to the manufacturer's instructions. mRNAs were prepared using the Oligotex mRNA Quiagen kit, the purification process being repeated once to eliminate any contamination. RNA concentration was determined by OD measurement (260 nm/280 nm), purity and integrity were assessed using an Agilent 2100 Bioanalyser.

### Microarrays and data analysis

Microarrays were obtained from the Service de Genomique Fonctionnelle (CEA, Evry, France). Two types of DNA collections were used for preparing the cDNA arrays: a collection of 5760 full length cDNA clones from the human infant brain 1NIB library, kindly provided by Genethon, and a collection of 2304 human PCR products (400–600 bp) corresponding to specific key-words selected by direct query in the Unigene database. A complete description of the microarrays used in this study, including the protocols for slide production, has been submitted to the GEO database [24] under accession number GPL4263.

For each microarray experiment, 20 μg of total RNA were reverse transcripted and indirectly labelled using the FairPlay Microarray Labelling Kit (Stratagene). Amino reactive Cy3- and Cy5- dyes (Cy™ Dye Post-Labelling Reactive Dye Pack, Amersham) were then chemically bound to cobalt and control cDNA respectively. Reverse labelling (or dyeswap) was performed for each experiment. Microarrays were pre-treated for 20 min with an N-methyl-pyrrolidinone solution containing 20 g/L succinic anhydride and 20 mM sodium borate, pH 8, immersed quickly in ethanol and dried by centrifugation for 6 min at 500 rpm. Following a quick rinse in RNase-free water, the slides were prehybridized in 30 ml 5X SSC, 1% SDS, 1% BSA (w/v) at 50°C for 40 min. The microarrays were then rinsed in RNAse-free water, immersed quickly in isopropanol and dried by centrifugation for 6 min at 500 rpm. The hybridization solution was prepared using 100 μl formamide, 10 μl SDS 10% and 30 μl RNase-free water. Labelled cDNA was solubilized in 17.5 μl of this solution plus 7.5 μl of 20 × SSPE and heat-denatured. Hybridization was performed overnight at 42°C. The microarrays were quickly rinsed in 0.1 × SSC, 0.01% SDS, washed twice for 10 min in 0.01 × SSC, then dried quickly in a stream of nitrogen and scanned with GenePix 4000B (Axon Instrument Inc., Forster City, CA). Cy3 and Cy5 spot fluorescence intensities were quantified after local subtraction of background using Genepix Pro 4.0 software (Axon Instrument Inc.). For each time point the result files were submitted to GeneSpring software 6.2 (Agilent Technologies) as follows. The data were first converted to take into account the results of the dyeswap reverse labelling, then normalized using the Lowess method, applying robust locally-weighted regression to smooth the intensity-dependence of the log ratios. The normalized data were then filtered on a quality test basis. This involved selecting spots detected on at least half of the microarrays with at least 70% pixels above threshold intensity (set to the median background plus two standard deviations).

From these remaining spots, we selected those with fluorescence ratios (representing cobalt-treated cells versus control) above 1.5 fold with p-value < 0.05 using a t-test statistical analysis on Genespring software and performing a Benjamini and Hochberg false discovery rate multiple testing correction.

### Quantitative real-time polymerase chain reaction (qRT-PCR)

Specific primers were designed with Primer3 [54] using cDNA sequences spotted on microarrays, and amplicons were controlled using Mfold [55]. The list of primers is given in table 3. Before differential analysis, good primer efficiency (80% -120 %) was checked as a mandatory test. One μg of total RNA (for 30 min, 2 h and 4 h) or 50 ng of mRNA (for 24 h) were reverse transcribed with oligo dT (12–18) using Omniscript reverse transcriptase (Qiagen) following the manufacturer's instructions. cDNA was diluted at least 10-fold with DNase-free water to 10 ng/μL. PCR was performed on 15 μl using a DyNAmoTM HS SYBR Green qPCR Kit (Finnzymes) on a VWR DNA Engine Opticon^® ^2 system. The amplification program consisted of 1 cycle at 95°C with 10 min-hold followed by 40 cycles at 95°C with 15 sec-hold, 60°C with 1 min-hold, and a reading step at 60°C for 1 sec. Amplification was followed by melting curve analysis between 65°C and 95°C. RNA was used as a negative template for the absence of residual genomic DNA and a negative control without cDNA was used to control overall specificity. House- keeping genes, which are generally used as reference genes, are often modulated by stress. For example, GAPDH is strongly induced by cobalt (ratio 3). To find an invariant gene at each time point, several candidates were selected for their invariance from all the microarray data, and tested as invariant genes in qRT-PCR. FIGF (c- fos induced growth factor) was selected for time points 0.5 h, 2 h and 4 h. For samples at 24 h, mRNA had to be purified from total RNA to detect TUBA3 as an invariant reference gene. Differential analysis was performed on cDNA templates obtained from cobalt-treated or untreated cells in sextuplets for each gene, in the same way as for the reference gene (5 ng for cDNA from total RNA or 0.1 ng for cDNA from mRNA). The results were processed using REST-MCS software [56] and tested for significance using a Pair Wise Fixed Reallocation Randomized Test by calculating a p value. Modulation was considered to be significant when p calculated by REST software was < 0.05.

### Western blot

Western blots were performed as previously described [53] after 24 h of cobalt exposure. Cells were lysed with 7 M urea, 2 M thiourea, 4% CHAPS, 20 mM spermine base, anti proteases (Roche cocktail), 40 mM DTT or 50 mM Tris-HCl, NaCl 150 mM ph7.5, 20 mM spermine base, anti proteases, 0.5% NP40 and 1% DOC. 10 to 50 μg of proteins were loaded onto a 4–12% or 12% NuPAGE gel in a MOPS or MES buffer (Invitrogen). Primary antibodies were: mouse monoclonal anti-DLG1 (sc-9961, 1/100, Santa Cruz), rabbit polyclonal anti-hsp90β (Ab1, 1/1666, NeoMarkers), mouse monoclonal anti-βactin (AC-15, 1/2000, Sigma), or mouse monoclonal anti-ubiquitin (P4D1, 1/400, Santa Cruz). A VECTASTAIN ABC kit (Vector laboratories) or a goat anti-mouse IgG coupled to HRP (1/10000, Novagen) was used for detection.

### Immunoassays

Immunoassays were performed on culture supernatants from cells exposed to various concentrations of cobalt over a 24 h period. The supernatants were tested crude or at 10 fold concentration. TIMP2 and FASL proteins were detected using ELISA kits (Raybiotech and R&D systems respectively) according to the manufacturer's instructions. The WFDC2 protein, was analysed in Dr Hellström's laboratory because they developed the WFDC2 ELISA [46]. Other immunoassays were performed by the CERBA laboratory (Cergy Pontoise, France).

## Authors' contributions

VM designed the study and drafted the manuscript.

FB carried out data acquisition and analysis and prepared the microarray results for tables and figures.

OP was involved in the design stage, qRT-PCR analysis and microarray experiments.

SR carried out qRT-PCR.

GS carried out the RNA extraction, labelling and microarray hybridization.

EQ was involved in the design stage and revised the manuscript

All authors read and approved the final manuscript.
